# Annual Research Review: Growth connectomics – the organization and reorganization of brain networks during normal and abnormal development

**DOI:** 10.1111/jcpp.12365

**Published:** 2014-12-01

**Authors:** Petra E Vértes, Edward T Bullmore

**Affiliations:** 1 Behavioural and Clinical Neuroscience Institute, Department of Psychiatry, University of CambridgeCambridge, UK; 2 Cambridgeshire and Peterborough NHS Foundation TrustCambridge, UK; 3 ImmunoPsychiatry, Alternative Discovery and Development, GlaxoSmithKlineCambridge, UK

**Keywords:** Brain networks, connectomics, development, cognitive change, neurodevelopmental disorders

## Abstract

**Background:**

We first give a brief introduction to graph theoretical analysis and its application to the study of brain network topology or connectomics. Within this framework, we review the existing empirical data on developmental changes in brain network organization across a range of experimental modalities (including structural and functional MRI, diffusion tensor imaging, magnetoencephalography and electroencephalography in humans).

**Synthesis:**

We discuss preliminary evidence and current hypotheses for how the emergence of network properties correlates with concomitant cognitive and behavioural changes associated with development. We highlight some of the technical and conceptual challenges to be addressed by future developments in this rapidly moving field. Given the parallels previously discovered between neural systems across species and over a range of spatial scales, we also review some recent advances in developmental network studies at the cellular scale. We highlight the opportunities presented by such studies and how they may complement neuroimaging in advancing our understanding of brain development. Finally, we note that many brain and mind disorders are thought to be neurodevelopmental in origin and that charting the trajectory of brain network changes associated with healthy development also sets the stage for understanding abnormal network development.

**Conclusions:**

We therefore briefly review the clinical relevance of network metrics as potential diagnostic markers and some recent efforts in computational modelling of brain networks which might contribute to a more mechanistic understanding of neurodevelopmental disorders in future.

## Introduction

The first decades of life are a time of enormous changes in the cognitive, social, sexual and economic repertoires of most humans. We normally progress from helpless infancy to independent adulthood in this period, which is also the time window for the highest rates of incidence of many psychiatric disorders (Paus, Keshavan, & Giedd, 2008). Such critical changes in normal adaptive behaviour, and risk for symptomatic or maladaptive behaviour, are presumably related to radical developmental changes in structure and function of the human brain. However, although we already have a fairly secure understanding of some microscopic processes and macroscopic markers of normal human brain development (Collin & van den Heuvel, 2013), it is currently much less clear how brain changes relate to behavioural changes over the course of life from birth to early adulthood (approximately 25 years old). It is also not yet clear how the high rates of emergence of psychiatric disorders in childhood and adolescence could be linked to the normative brain developmental processes ongoing at the same time.

One strategy that may help us to address these key questions in future is to focus especially on the organization (and reorganization over developmental time) of brain networks. The network perspective is likely to be informative for two convergent reasons. First, we already know that changes in synaptic connectivity and axonal myelination are among the key microscopic processes in postnatal development (Collin & van den Heuvel, 2013); and that changes in cortical thickness and white matter volume are among the most well-replicated magnetic resonance imaging (MRI) markers of macroscopic development (Giedd & Rapoport, 2010). It seems reasonable to expect that this prior knowledge should be compatible with developmental change in the structural networks of interactions between cells (or regional populations of cells) at micro (or macro) scales. Second, we also know that ‘higher-order’ cognitive processes that will be fundamental to successful adult independence – such as planning, problem solving, working memory – are not phrenologically localized to a specific brain region. Instead, most consciously effortful cognitive processes are thought to emerge from large-scale networked interactions between multiple regional populations of cells that may be anatomically separated from each other over long distances (Dehaene, Kerszberg, & Changeux, 1999). Thus, it seems reasonable to expect that the broad behavioural changes of normal childhood and adolescence should be accompanied by major changes in anatomical and functional brain networks.

## Background and scope

### Brain graphs and growth connectomics

In what follows, we will review studies from the recently growing literature on brain network development. We will focus especially on so-called ‘growth connectomics’: studies that have used graph theoretical measures to analyse the development of brain networks. Graph theoretical models of a brain network are typically quite simple (Bullmore & Sporns, 2009): a collection of nodes interconnected by edges. At a microscale, the nodes could be neurons and the edges could by synapses; at the macroscale of neuroimaging, the nodes are brain regional volumes in order of mm and the edges are typically thresholded statistical measures of structural or functional connectivity between regions.

Such graphical models, or brain graphs, can be used to estimate topological network properties, such as small-worldness or resilience to attack; as well as spatial network properties, such as the distance in mm between connected nodes. Importantly, graph theory is sufficiently general to encompass network analysis over a wide range of neuroscientific modalities – from multielectrode array recordings of neuronal cultures in vitro to functional MRI recordings of whole-brain resting state dynamics in vivo. Thus, graphs create an opportunity to use the same mathematical language to quantify aspects of brain network development at micro- and macrolevels of measurement. This generalizability of graphical analysis across scales of time and space may make it easier to identify aspects of brain network organization that are scale invariant, or consistently expressed at both micro- and macroscales; and thus to make more secure linkages between cellular processes and imaging markers of brain development.

**Figure 1:**
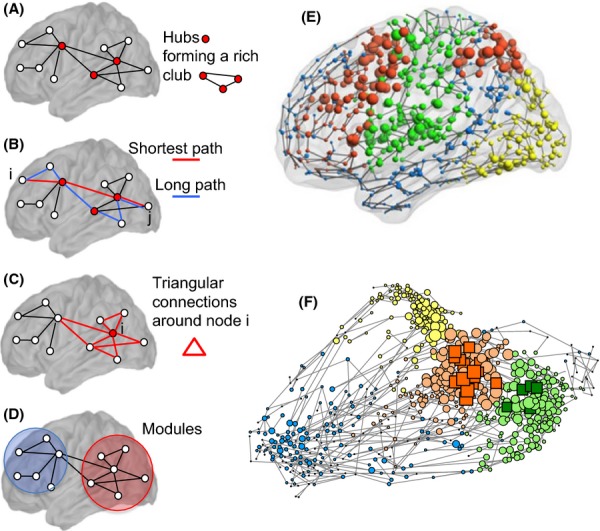
Network metrics commonly used to quantify the topology of networks. (A) Degree, hubs and rich clubs: The degree of a node is the number of links or connections it has to the rest of the network. In many real-world networks however, including brain networks, the degree distribution has a fat tail weighted towards high degrees. This means that most nodes only have a few connections, but a few nodes are extensively connected. High-degree nodes are often referred to as hubs or as ‘rich’ nodes. In many networks, these nodes have a tendency to preferentially connect to one another, forming an elite group of nodes called a rich club (Zhou & Mondragón, 2004). (B) Path length and efficiency: The minimum path length between two nodes i and j is simply the minimum number of edges that need to be traversed from one node to another. In contrast to regular lattices, real-world networks tend to have low average path length, facilitating the flow of information between distant parts of the network. Efficiency is inversely related to path length but has the advantage of being well defined even in networks that contain some disconnected elements (Latora & Marchiori, 2001) Networks for which the average path length from one node to another is small can thus be said to have high global efficiency. (C) Clustering and local efficiency: The clustering coefficient measures the number of connections that exist between the first neighbours of a node i as a proportion of the maximum possible number of such connections. These edges form triangular connections around the node i which increase the so-called local efficiency by decreasing the path length locally between the node's neighbours. (D) Modularity: Many complex networks have a modular community structure, whereby they contain subsets of highly interconnected nodes called modules. Various algorithms have been designed to find such modules in real-world networks, typically by maximizing the fraction of the network's edges that are intramodular rather than intermodular (Newman, 2004). The identification of modules is of particular interest in biological networks as it may uncover functional units in data that are otherwise difficult to interpret. Many of these features are highlighted in a brain functional network shown in both anatomical space (E) and topological space (F). Node size is proportional to degree and rich club nodes are highlighted as squares in (F). The modular organization is highlighted by assigning different colours to nodes of different modules. (Adapted from Crossley et al., 2013, with permission.)

Indeed, it has been remarkable to note, as network science has gathered momentum in the last 15 years or so, that both macro- and microscale brain networks as well as many other complex systems – from social networks to the internet – share certain key organizational principles (Figure1; Bullmore & Sporns, 2009). One well-known example of widely shared organizational structure is the small-world phenomenon, whereby networks are simultaneously highly clustered (nodes that are connected to each other are also likely to have many nearest (first degree) neighbours in common) and highly efficient (the average path length between a pair of nodes is short). This small-world architecture was originally described in the context of the collaboration graph of film actors, the power grid of the western United States, and the neuronal network of the nematode worm *Caenorhabditis elegans* (Watts & Strogatz, 1998). The same structure has since been consistently found in numerous empirical studies of structural and functional brain networks in humans and other animals (Bullmore & Sporns, 2012).

Hubs are also found in almost all complex networks. Hubs can be defined in many ways, but the simplest definition is as high-degree nodes, where the degree of a node is the number of edges that connect it to other nodes in the network. High-degree hub nodes, with many connections to the rest of the network, are more probable in internet, metabolic, social and brain networks than expected in a random graph: the degree distribution is more fat-tailed than it would be for a random graph. For example, the degree distribution of hubs in human brain networks is typically a power law that is exponentially truncated at a cut-off maximum number of edges (Achard, Salvador, Whitcher, Suckling, & Bullmore, 2006; Barabási & Albert, 1999). Many complex networks share a further related property – the tendency for the hubs to connect to each other to form a rich club of hub nodes that are densely connected to each other and to the rest of the network (Zhou & Mondragón, 2004).

Another ubiquitous feature of network organization is modularity. Much like companies, brain systems tend to be organized in modules with a high level of interaction within modules and sparser connectivity between them. This near-decomposability of systems into quasi-independent units has well-known operational advantages (Simon, 1962). Modules also tend to be hierarchically organized and are spanned by a number of highly connected nodes, or hubs (Figure1A). Here too, the intuitions based on other complex systems – such as the organization of social structures – hold true for brain networks: hubs often hold privileged positions of functional importance (integrating information from various parts of the brain), but are also points of vulnerability, as attacks targeting these nodes will rapidly compromise the system as a whole (Albert, Jeong, & Barabási, 2000; Barabási & Albert, 1999; de Solla Price, 1965).

### The economics of brain network organization

As it has become clearer that nervous systems generally have nonrandom topological properties of the same kind as seen in many other complex systems, it has become more important to understand what might account for the near-universality of features like small-worldness, hubs, modules, rich clubs, etc. One way of thinking about this question is to ask: what kind of selection pressures would favour emergence of brain network topology? And is it likely that the same selection pressures would also favour emergence of network topology in a nearly universal class of spatially embedded systems for information exchange?

Since the 19th century work of Ramón y Cajal and others, it has been recognized that one very important selection pressure on brain network formation is cost. The brain is biologically expensive. For example, although accounting for only 2% of body mass, the brain consumes about 20% of the body's metabolic budget. This is despite many aspects of brain organization being nearly minimized for cost (Chen, Hall, & Chklovskii, 2006; Niven & Laughlin, 2008). For example, as predicted by Cajal's conservation principle, most of the anatomical connections between neurons (in any nervous system) are short distance, because the wiring cost generally increases as a function of distance. However, it has been shown by computational models that wiring cost minimization alone does not reproduce the full repertoire of topological features that are seen in naturally selected nervous systems.

Cost-minimized networks will not include any long-distance connections – they will therefore tend to a lattice-like topology. Low cost, lattice-like networks have high clustering and modularity, and high resilience to random attack, all of which are hallmarks of real brain networks; but they do not have high-degree hubs (in a perfect lattice all nodes have identical degree), rich clubs or high global efficiency (short path length).

For example, in the *C. elegans* nervous system, it has been shown that further reductions of connection distance may be achieved by rewiring the biological network in silico, but only at the expense of increasing the path length (or decreasing the global efficiency) of the network (Kaiser & Hilgetag, 2006). Indeed, many topologically integrative properties that have been empirically observed in brain networks – such as efficiency or rich clubs – depend crucially on the existence of some long-range links spanning large anatomical distances. These high-cost network features have been associated with benefits to integrated information processing both theoretically and to some extent empirically. Topological efficiency of human brain networks, for example, has been positively correlated with IQ (van den Heuvel, Stam, Kahn, & Hulshoff Pol, 2009; Li et al., 2009) and improved attentional task performance (Kitzbichler, Henson, Smith, Nathan, & Bullmore, 2011). Rich clubs of highly interconnected hubs (command interneurons) in *C. elegans* are functionally important for the organism's coordinated movement and adaptive behaviour (Towlson, Vértes, Ahnert, Schafer, & Bullmore, 2013). Rich clubs of high-degree cortical hubs in the human fMRI coactivation network are analogously important for ‘higher-order’ executive functions such as planning and working memory (Crossley et al., 2013). The generalization that emerges is that brain organization, at all scales, may be shaped by competition or trade-off between (at least) two selection pressures: cost and integrative topology. An economical formulation of this trade-off is in terms of a selection pressure to minimize biological cost contending with a pressure to maximize topological value. As some of the most adaptively valuable functions of the brain depend on its integrative network capacity, the extra cost of hubs, clubs and intermodular connections is presumably a biological price worth paying.

It is important also to note that the optimal outcome of such trade-offs between competitive natural selection criteria will not be fixed over developmental time. Most obviously the configuration of functional networks must be dynamically variable as environmental contingencies and demands for cognitive processing vary from moment to moment. The connectomics literature to date has mainly focused on the time-averaged organization of functional networks measured during nominally constant experimental conditions. For example, fMRI networks have typically been constructed from correlational analysis of several minutes of time series data recorded during the ‘resting state’. Recent studies have begun to demonstrate that changes in network configuration occur spontaneously while subjects lie quietly at rest (Chang & Glover, 2010) and also when task-related demands for cognitive processing are manipulated experimentally (Kitzbichler et al., 2011). We anticipate that configuration of brain networks, both structural and functional, will also prove to be dynamically variable over the longer timescales of maturation and senescence. Specifically, we expect that economical trade-offs between topological value and biological cost will be continuously renegotiated over the course of brain network growth.

### Some stylized facts about brain development

**Figure 2:**
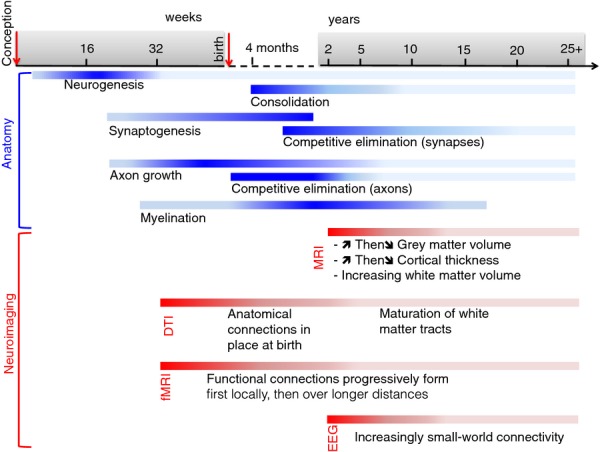
Gantt chart of the sequence of events in brain maturation (blue) and related neuroimaging observations (red) (Collin & van den Heuvel, 2013; Giedd & Rapoport, 2010). The intensity of colour in each bar corresponds to the intensity of developmental changes observed

To contextualize the more recent, and still incomplete, results of growth connectomics, we will first rehearse some ‘stylized facts’ about human brain development that are more certainly known at this time (see Figure2).

The bulk of neurogenesis and neuronal migration is thought to be complete by about week 20 after conception (Stiles & Jernigan, 2010) at which point axons begin to form and the density of synapses increases steadily (by about 4% per week) until week 27 after conception (Collin & van den Heuvel, 2013). This developing cytoarchitecture has also been suggested to determine the concomitant increases in the degree of cortical folding as well as the development of individual patterns of sulco-gyral folding (Ronan et al., 2013).

The period leading up to birth is characterized by an exuberant increase in axonal growth (Innocenti & Price, 2005). At birth, the human brain is relatively large, complexly folded, and highly active metabolically. Immediately following birth, brain development goes through a further proliferative phase, during which the number of synaptic connections increases markedly. This peak in synaptogenesis gives way to a consolidation phase characterized by monotonically decreasing numbers of cortical neurons, axonal connections and synapses (at a microscale). This process of cortical neuronal cell loss and synaptic pruning is approximately synchronized with progressively increasing myelination of white matter tracts (Collin & van den Heuvel, 2013).

These key microscopic processes of the consolidation phase (cell loss, synaptic pruning, myelination) are broadly convergent with developmental changes in macroscopic measurements made by neuroimaging. MRI studies have consistently shown decreasing grey matter volume and cortical thickness, and increasing white matter volume, both over the age range 7–24 years, approximately. In particular, longitudinal MRI studies (with more than one measurement per subject between the ages of 4–22 years) have shown increases in white matter volumes and inverted U-shaped trajectories of grey matter volumes with different regions of grey matter reaching maximal volumes at different times (Giedd & Rapoport, 2010; Giedd et al., 1999; Gogtay et al., 2004). In addition, diffusion tensor imaging (DTI) studies have documented the maturation of major white matter fibre tracts as well as age-related changes in white matter diffusion properties, with anisotropy increasing and overall diffusivity decreasing with age due to increasing myelin and myelin maturation [for review see (Cascio, Gerig, & Piven, 2007; Collin & van den Heuvel, 2013)]. These processes were found to be most prominent in early childhood until the age of 2, but more recent studies have shown they continue into late adolescence (up to 18 years) and possibly early adulthood (up to 32 years; Giorgio et al., 2008; Hagmann et al., 2010; Lebel & Beaulieu, 2011; Snook, Paulson, Roy, Phillips, & Beaulieu, 2005).

### Recent reviews of brain network development

A number of recent review papers have focussed on the developing human connectome, with varying degrees of emphasis on the areas covered in this paper. Power, Fair, Schlaggar, & Petersen (2010), Vogel, Power, Petersen, & Schlaggar (2010) and Menon (2013) offered comprehensive overviews of the development of human brain functional networks. In contrast, Hagmann, Grant, & Fair (2012) reviewed the development of both structural and functional networks as well as the development of structure-function coupling; and provided an insightful overview of the remaining technical limitations and open biological issues related to developmental network studies. Finally, Collin & van den Heuvel (2013) provided a chronological account of brain network development from prenatal connectomes to adulthood and ageing, with a stronger emphasis on underlying neurobiological processes and less separation between functional and anatomical network findings.

In this review, we extend these previous accounts of brain network development, integrate results from all macroscopic neuroimaging modalities, include insights gained from the growth of microscopic neuronal networks, and emphasize in particular the potential driving forces shaping brain networks into their observed organization. Given this broad scope, the current review is necessarily selective. It aims to provide a balanced and contextualized synthesis of the literature on graph theoretical analysis of brain networks during development to stimulate further research in growth connectomics.

## Normative development of brain networks

### Macroanatomical networks

Large-scale brain anatomical networks describe the ‘wiring diagram’ of the whole brain, or a large part of it, showing how different cortical and subcortical regions (in the order of mm volume) are interconnected by white matter tracts or fascicles consisting of large numbers of parallel axonal projections. Neuroimaging methods have provided most of the available data on anatomical network development at this scale. Conventional MRI scans can be analysed to estimate patterns of interregional covariance or correlation in grey matter volume, cortical thickness or longitudinal trajectory: these approaches are collectively known as structural covariance analysis and provide a network perspective based on multiple local grey matter measurements (Alexander-Bloch, Giedd, & Bullmore, 2013). Diffusion tensor imaging (DTI) and related techniques provide data that can be analysed to estimate the probability of an axonal tract connecting two regions of grey matter. We will refer to these approaches collectively as providing a network perspective based on anatomical data.

#### White matter tractography networks (DTI)

In DTI, each voxel of the brain is associated with a tensor representing the rate of water diffusion along different directions at that point in space. This information can also be summarized by the metric of fractional anisotropy (FA), which is a scalar value between zero and one describing the degree of anisotropy of the diffusion process at every voxel. The arrangement of the axons in parallel bundles tends to facilitate the diffusion of the water molecules preferentially along their main direction thus increasing the value of FA.

Fractional anisotropy is therefore thought to reflect fibre density, axonal diameter, and myelination in white matter, but this complex measure inevitably combines several poorly understood biophysical properties of white matter. Simpler metrics such as the axial and radial diffusivity alone may therefore prove more tractable in terms of linking diffusion metrics to underlying tissue microstructure. For example, myelination has been shown to play a particularly important role in radial diffusivity (Song et al., 2002).

In addition to computing FA, the principal direction of the diffusion tensor can be used to track white matter fibres along their length. Such tractography algorithms are therefore able to infer the patterns of white matter connectivity between predefined grey matter regions of interest (ROIs) across the whole brain (Hagmann et al., 2007). Depending on the resolution chosen when defining the ROIs, this procedure results in a network – or connectome – with anywhere between tens and thousands of nodes connected by edges representing white matter fibres. These edges are either considered to be binary (Tymofiyeva et al., 2013; Yap et al., 2011) or weighted by the number (van den Heuvel, Kahn, Goñi, & Sporns, 2012; Lim, Han, Uhlhaas, & Kaiser, 2013) or density (Hagmann et al., 2007) of fibres. More recently, connection strengths have also been weighted by diffusion-derived parameters such as the inverse of the average apparent diffusion coefficient (ADC) measured along the entire length of the same pathway (Hagmann et al., 2010).

Despite such differences in methodology, diffusion imaging studies have yielded convergent results on the broad topological features of both adult and developing brain networks. The adult connectome was found to be small-world and modular, with most connections linking regions that are spatially close and functionally related, and fewer long-range connections ensuring high global efficiency of the network (Bullmore & Sporns, 2009). These networks were also shown to exhibit a fat-tailed degree distribution, with hub nodes typically identified among parietal and prefrontal regions, and with the precuneus and insula consistently highlighted as playing a central topological role [see (Bullmore & Sporns, 2009) and references therein].

A range of paediatric studies have shown that many of the broad topological features of the human DTI connectome – such as small-worldness or hubs – are already established at birth. This is consistent with neurobiological evidence of axonal growth cone markers falling off sharply after birth (Benowitz & Routtenberg, 1997) and with the observation that all major white matter pathways are present in newborns (Dubois, Hertz-Pannier, Dehaene-Lambertz, Cointepas, & Le Bihan, 2006; Liu et al., 2010; Collin & van den Heuvel, 2013; Hagmann et al., 2012). The prominence of these existing topological features, however, is set to evolve gradually in the first few years of life, reflecting other major neurodevelopmental processes in action over this period (Paus, 2010).

Over the course of normal development, human DTI networks gradually mature from local, proximity-based connectivity patterns designed to support primary functions to a more distributed, integrative topology (Collin & van den Heuvel, 2013; Tymofiyeva et al., 2013; Yap et al., 2011) thought to be favourable for supporting higher cognitive functioning (Bullmore & Sporns, 2012; Collin & van den Heuvel, 2013; Hagmann et al., 2010; van den Heuvel et al., 2012). Although the major modules of the structural connectome are also relatively stable from birth, the connections between these modules are markedly increased as long fibre pathways linking them together mature (Hagmann et al., 2010). Correspondingly, measures of integration such as global efficiency have been found to increase continuously both during infancy (first 2 years of life; Tymofiyeva et al., 2013; Yap et al., 2011) and in the age range 2–18 years (Hagmann et al., 2010). In contrast, measures of local efficiency such as clustering have been shown to decrease over the same period (Hagmann et al., 2010; Tymofiyeva et al., 2013; Yap et al., 2011). These trends were also replicated in a recent study investigating later adolescence and early adulthood (12–30 years) which found significant decreases in path length, clustering, small-worldness and modularity of whole-brain networks with age, plateauing in early adulthood (Dennis et al., 2013).

Interestingly, in spite of these large-scale changes in the integrative capacity of anatomical networks over time, the hubs of the network appear to be largely fixed by the age of 2 (Hagmann et al., 2010) and only functional MRI studies highlight the continued evolution of their role in infants (Fransson, Åden, Blennow, & Lagercrantz, 2011) and potentially up to, but not beyond, early childhood (Hwang, Hallquist, & Luna, 2012).

The remodelling of anatomical networks over the course of postnatal development is thought to predominantly reflect the fact that myelination and myelin maturation occur asynchronously across various axonal tracts (Hermoye et al., 2006). For example, axonal maturation has been shown to occur over longer timescales in association cortex than in primary cortical regions (Glasser & Van Essen, 2011; Lebel et al., 2012). Most recently, it has also been shown that while the number of fibre tracts remains largely stable between 4 and 40 years of age, there is a substantial overall decrease in the number of streamlines within a given fibre tract, affecting in particular those tracts that are short, within modules, within hemispheres and have a large number of streamlines to begin with (Lim et al., 2013).

Unfortunately, not all known neurodevelopmental processes are appropriately captured in tractography data. Synaptic density, for example, is known to peak around 1 year of age, followed by extensive synaptic pruning in early childhood (Huttenlocher, 1984). Before the age of 2, this decrease in microscopic connections is known to be accompanied by a corresponding decrease in long-range axonal connections and axonal branching (Low & Cheng, 2006; Luo & O'Leary, 2005). However, DTI studies over the same age range typically show an increase in the number of tracts, likely reflecting an artefact of tractography (Hagmann et al., 2012) which obscures this early underlying biological process. Importantly, by the age of 2, the pattern of macroscopic axonal connections is thought to be relatively constant (Low & Cheng, 2006; Luo & O'Leary, 2005). Thereafter, until late adolescence and early adulthood, the remaining connections will both increase in diameter and become gradually myelinated, giving rise to wide-spread changes in white matter structure and composition, and these later developmental processes have been more accurately captured by anatomical networks derived from diffusion imaging (Giedd et al., 1999; Hagmann et al., 2010; Huang et al., 2006; Paus, 2010).

#### Grey matter covariance networks (MRI)

In addition to diffusion imaging, brain structural networks have also been mapped using cross-correlations in morphological metrics, such as cortical thickness or volume, measured in conventional MRI data on large groups of individuals (Lerch et al., 2006; Rockel, Hiorns, & Powell, 1980; White et al., 1997; Wright et al., 1999). A limitation of this approach – so-called structural covariance network analysis – is that it results in a single network for the entire group of subjects under study. It is thought nevertheless to encode biologically meaningful information about the average structural connectivity between brain regions. For example, large-scale patterns of covariance in cortical thickness or volume have been found to be statistically robust, anatomically plausible and heritable (Alexander-Bloch, Raznahan, Bullmore, & Giedd Bullmore, 2013; Schmitt et al., 2008). Biologically, such structural covariances between pairs of brain regions are thought to reflect direct connections that mediate mutually trophic and other effects which result in the observed synchronization of maturational changes between regions (He, Chen, & Evans, 2007; Lerch et al., 2006; Mechelli, Friston, Frackowiak, & Price, 2005; Raznahan et al., 2011; Zielinski, Gennatas, Zhou, & Seeley, 2010).

Network analysis of structural covariance networks revealed many of the same topological properties as found in DTI networks including small-worldness and modularity (He et al., 2007; Chen, He, Rosa-Neto, Germann, & Evans, 2008; Bassett et al., 2008). Some studies suggest remarkable convergence between structural covariance and these other measures of brain connectivity, in the composition of large-scale modules and even the strength of individual edges (Chen et al., 2008; Kelly et al., 2012; Seeley, Crawford, Zhou, Miller, & Greicius, 2009). However, there is evidence that this overlap, while significant, is most likely incomplete, that is, some interregional correlations in cortical anatomy measured using MRI can be observed even in the absence of tractographic evidence of direct anatomical connections from DTI (Alexander-Bloch et al., Raznahan, & Bullmore, 2013; Gong, He, Chen, & Evans, 2012). The exact interpretation of such discrepancies is currently an open question. On the one hand they may indicate that structural covariance between a pair of cortical regions can in fact arise in the absence of a direct axonal connection between them. On the other hand it is possible that DTI tractography simply underestimates the number of axonal connections between structurally covariant regions.

More recently, these results based mainly on groups of healthy adults have been extended to paediatric groups and longitudinal studies of development. One longitudinal study of 28 healthy paediatric subjects, collected at 1 month, 1 year and 2 years showed that small-world and modular topology was already present at 1 month in networks derived from morphological correlations of brain regional volumes (Fan et al., 2011). The network's global efficiency was shown to be further increased between 1 and 2 years as well as from year 2 to adulthood. In contrast, initial increases in modularity and local efficiency observed between year 1 and year 2 were not seen to extend beyond the age of 2 years in this study. Mostly, convergent results were also found by a study of structural brain networks constructed from cortical thickness measurements in four older age groups [early childhood: 4.8–8.4 years (y); late childhood: 8.5–11.3 y; early adolescence: 11.4–14.7 y; late adolescence: 14.8–18.3 y]. Khundrakpam et al., (2012) showed a significant shift in topological properties including decreased modularity and clustering as well as increased global efficiency and increased number of connector hubs during late childhood. Another study that measured structural covariance of a single ‘seed’ region with the rest of the network in these same age groups made an interesting distinction between (developmentally integrated) brain areas whose covariance with the rest of the brain increased monotonically, and (developmentally segregated) brain areas whose covariance with the rest of the brain peaked in early adolescence before contracting in late adolescence (Zielinski et al., 2010).

Subsequent longitudinal studies have tightened the explanatory link between structural covariance and coordinated development of pairs of brain regions (Alexander-Bloch et al., 2013; Raznahan et al., 2011). Longitudinal data on 108 individuals aged 9–22 years at enrolment (with 3–6 longitudinal scans on each participant over 6–12 years of follow-up) were used to construct maturational networks based on correlations between various brain regions’ rates of cortical thinning. It was shown that, on average, the thickness of the cortex shrinks by about 10% from an initial thickness of 3.3 mm at 9 y to a stable adult thickness of about 3 mm by 22 y, but different brain regions differed subtly from each other in terms of the trajectory of cortical thinning within the same subject; and subjects also differed from each other in their individual thinning trajectories. Alexander-Bloch et al. estimated the synchronization of cortical thinning trajectories between each pair of cortical nodes and used graph theory to analyse the pair-wise synchronization matrix as a brain maturational network. These maturational networks were shown to share a range of global, nodal and local, so-called mesoscopic, topological properties with structural covariance networks constructed from the same subjects. In addition, maturational and structural connectivity were found to be highly correlated across brain regions. This was interpreted to indicate that brain structural covariance networks reflect synchronized developmental change in distributed cortical regions. In other words, regions which show similar developmental trajectories of cortical thinning – putatively related to synaptic pruning at a cellular scale – will tend to have positively correlated cortical thickness volumes over the adult population (Alexander-Bloch et al., 2013).

### Macrofunctional networks

#### Resting state fMRI functional connectivity networks

Functional MRI (fMRI) was originally used to determine the function of individual brain regions by measuring the blood oxygen level-dependent (BOLD) signal as a proxy of neuronal activity during various tasks. More recently, resting state fMRI recordings have also been used to understand the correlational structure of brain activity (Biswal, Yetkin, Haughton, & Hyde, 1995). Two brain regions that display highly correlated BOLD time series during rest are said to be ‘functionally connected’ even when they are not known to be physically connected by a direct anatomical pathway (Biswal et al., 1995; Fox et al., 2005; Greicius, Krasnow, Reiss, & Menon, 2003; Lowe, Mock, & Sorenson, 1998; Vincent et al., 2007). Such functional coupling between node pairs lacking direct anatomical linkage can be mediated by indirect structural connections or by other anatomical drivers of spatial auto-correlations in the fMRI signal (Hagmann et al., 2010; Honey et al., 2009).

As in anatomical networks, the ROIs chosen as the nodes of a functional network vary between studies in their location, size and number. Despite these differences, however, whole-brain functional networks in healthy adults (where the ROIs cover the entire cortical area) have been found to display a set of topological properties convergent both across fMRI studies and with the properties of anatomical networks, for example, small-worldness, hubs, modules and rich clubs (Bullmore & Sporns, 2009).

As early as 2007, statistical comparison of functional brain network organization between young and elderly adults found decreased topological efficiency in the older group (Achard & Bullmore, 2007). This suggests that normal processes of brain maturation might also give rise to quantifiable changes in functional network topology. However, most developmental studies to date have not focussed directly on the topological properties of such whole-brain networks (Power et al., 2010). Instead, they have most often studied seed-based maps which highlight on an anatomical image of the brain the areas whose activity is most correlated with that of a given ‘seed’ region of interest. From a network perspective, this corresponds to examining all the connections emanating from a single node. Other studies used independent component analysis (ICA) to decompose the BOLD signal and highlight groups of brain regions (sometimes called ‘resting state networks’ in the literature) whose activity contains the largest proportion of each of these independent signal components. From a network perspective, each of these spatiotemporal ICA components corresponds approximately to a module (or submodule) of the whole-brain functional network. Finally, some studies have constructed functional brain graphs, but restricted the nodes to a small number of ROIs corresponding to regions previously identified by activation mapping of task-based fMRI data. These task-defined sets of regions are also often called networks in the literature, for example, the central executive network, (CEN) involved in goal-directed behaviour; or the widely studied default mode network (DMN), involved in internally oriented cognition. The ROIs in such task-defined networks tend to be anatomically distributed. For instance, the DMN is composed of regions whose activity is known to decrease during goal-directed tasks, including bilateral precuneus, posterior cingulate, angular gyrus, inferior temporal, parahippocampal, superior frontal and medial prefrontal cortex regions (Raichle et al., 2001; Shulman et al., 1997). Clearly, seed-based correlation maps, ICA and regional networks are all conceptually somewhat related and taken together the results of these studies add up to a strong prior on the kind of topological changes we expect to see by graph theoretical analysis of whole-brain fMRI networks over the course of development (Power et al., 2010).

Numerous paediatric resting state fMRI studies have shown the existence of correlated spontaneous activity in infants and young children at various stages of development (Damaraju et al., 2014; Fransson et al., 2009; Gao, Alcauter, Smith, Gilmore, & Lin, 2014; Gao et al., 2009; Lin et al., 2008; Liu, Flax, Guise, Sukul, & Benasich, 2008); including premature infants scanned before and at term equivalence (Fransson et al., 2007; Smyser et al., 2010; Lee, Morgan, Shroff, Sled, & Taylor, 2013) as well as scans during foetal life (Thomason et al., 2013). Most primary sensory and sensorimotor systems are already functionally delineated at birth but longer range functional connections, particularly between anterior and posterior cortical regions, appear to be limited in infants under the age of 2 (Fransson et al., 2007; Smyser et al., 2010; Gao et al., 2011, 2014).

In older children (between the ages of 4 and 14), most studies have focussed on describing the state of functional connectivity within the default mode network. Studies examining the presence and maturity of the DMN in this age range, as well as the level of connectivity in anterior–posterior directions more generally, have thus far revealed imperfectly consistent results (Damaraju et al., 2014; Gao et al., 2014; Power et al., 2010). Despite disagreement on the particular age at which the DMN clearly emerges, however, all studies converged on a general trend of increased functional connectivity within the DMN over development from early childhood to adolescence. This trend was also reinforced by the observation, replicated over several studies, that cortical connections spanning short anatomical distances tend to be strong in infants and weaken over development, while long-range edges tend to strengthen over development from birth to early adulthood (Fair et al., 2007, 2009; Gao et al., 2011; Kelly et al., 2009; Qin, Young, Supekar, Uddin, & Menon, 2012; Supekar, Musen, & Menon, 2009). Interestingly, the opposite trend was observed in the context of subcortical–cortical connections which were also found to be extensively reconfigured over the period 7–22 years, with subcortical areas being more strongly connected to primary sensory, association and paralimbic areas in children than in adults (Supekar et al., 2009).

The first explicit use of graph theory in developmental studies of functional data focussed on 210 subjects between 7 and 35 years old and studied how the modular structure of a network comprising 39 task-defined ROIs changed over this age range (Fair et al., 2007). The study found that the cingulo-opercular and fronto-parietal components of the task control network, that are normally distinct in adults, were initially merged in children. Over the course of development, the functional networks gradually reorganized with anterior cingulate and prefrontal nodes splitting from other frontal nodes and becoming more tightly connected with insular and thalamic nodes. A second study of a network comprising 34 ROIs, from the default-mode, task control, and error-responsive networks, showed similar developmental changes in the composition of modules in the age range 8–25 years. In particular, modular organization shifted from a largely anatomical grouping of regions in childhood to a grouping based on functional role in young adults (Fair et al., 2009). Similar results have subsequently been reproduced using larger sets of regions derived from meta-analysis of multiple task-related data sets such as fMRI studies of speaking, button press and single word reading (Power, Cohen, Miezin, Schlaggar, & Petersen, 2009; Power et al., 2010; Vogel et al., 2009).

These changes in modular organization were also echoed by studies of degree and betweenness centrality during infancy (Gao et al., 2011), in infants versus adults (Fransson et al., 2011), and children versus adults (Uddin, Supekar, Ryali, & Menon, 2011). These showed that high-degree hubs coincide with regions of high betweenness centrality in both groups, but that the location of these hubs progressively shifts from areas near the primary sensorimotor cortex in infants to areas within the DMN in older children and adults. Note however that this shift was found to predominantly happen early on, with the location of hubs appearing stable between the ages of 10 and 20 years and the strength of functional connectivity of these hubs stabilizing during adolescence (Hwang et al., 2012). Overall, this developmental transition from local community organization to a distributed topology sits well with prior observations that the strength of short-range functional connections decreases, while long-distance connection strengths increase over development.

Despite this evidence for large-scale reorganization of brain functional network, however, the major global metrics have in general not been shown to change over the same period. Modularity – or the extent to which the network is separable into modules – was found to remain constant between children and young adults (8–25 years) even while the composition of the modules was found to change (Fair et al., 2009). Similarly, clustering and global efficiency were not found to change significantly from childhood to adulthood, whether in whole-brain or more limited functional networks (Fair et al., 2009; Fransson et al., 2011; Supekar et al., 2009). One recent study found significant increases in local and global efficiency as well as connection distance, but only between neonates and 1-year old infants, with the difference ceasing to be significant between years 1 and 2 (Gao et al., 2011). More recently, Wu et al. found increases in clustering and the related measures of local efficiency and small-worldness over the age range 6–18 years, but global efficiency was not found to change over the same period (Wu et al., 2013). These results were also echoed in recent work by Cao et al. (2014) who studied the age range 7–85 years and found local efficiency to increase slightly until about 25 years of age. They noted however that these results were not robust to the use of different parcellation templates.

At present, the most widely reproduced developmental result regarding fMRI connectivity is the gradual strengthening of long-range connections over time, at the expense of the shorter range local connections that dominate early on. This trend was observed both in infants (over the course of the first 2 years of life; Gao et al., 2011) and over the age range 7–35 as well as 8–25 years (Fair et al., 2007; Kelly et al., 2009; Supekar et al., 2009). Apart from the lack of evidence for developmental changes in global brain network topology, prior studies have either directly replicated this finding or were conceptually consistent with it. These developmental changes in the typical distances spanned by functional connectivity have even been used to make fairly accurate predictions of brain maturity at the level of individual subjects between 7 and 30 years of age (Dosenbach et al., 2010). Importantly, however, recent work has highlighted the vulnerability of resting state fMRI to the effects of in-scanner head motion and the critical role of careful preprocessing steps in mitigating these artefacts (Hallquist, Hwang, & Luna, 2013; Patel et al., 2014; Power, Barnes, Snyder, Schlaggar, & Petersen, 2012). Several techniques have subsequently been suggested to effectively deal with biases resulting from head motion (Bright & Murphy, 2013; Kundu et al., 2013; Patel et al., 2014), however concerns remain about many earlier findings related to differential effects of development on short versus long-distance connectivity estimates (Power et al., 2012; Satterthwaite et al., 2012, 2013; Van Dijk, Sabuncu, & Buckner, 2012; Yan et al., 2013). The field awaits follow-up studies, with larger sample sizes and state-of-the-art controls for potential motion-related artefacts, to clarify whether the apparently well-replicated shift from short to long-distance connectivity in developmental fMRI is a reliable marker of underlying biological processes; or a measurement bias induced by the greater amount of small, transient head movements expected during several minutes of fMRI scanning of younger children.

#### Electrophysiological (EEG, MEG) networks

Human brain functional networks can also be constructed from electrophysiological recordings (EEG, MEG) which provide complementary insights into correlated neurophysiological dynamics and functional networks across the brain. In contrast to fMRI, these methods measure neuronal activity directly and have excellent temporal resolution with bandwidths typically of 1–100 Hz (compared to the 0.001–0.5 Hz range accessible to fMRI). This is especially valuable as both very low and very high frequency oscillations have been shown to have neurophysiological significance (Bragin, Engel, & Staba, 2010; Van Someren, Van Der Werf, Roelfsema, Mansvelder, & da Silva, 2011). The trade-off, however, is that EEG and MEG have worse spatial resolution (on the order of millimetres or centimetres) and the signal is not recorded at its anatomical source but rather at surface sensors, which complicates both the interpretation of results and the separation of true signal from so-called volume conduction effects spreading each signal source across several sensors.

Despite these differences, electrophysiological recordings can also be used to construct robust networks representing resting state functional connectivity based on similarity or synchronization of the signal at various sensors. Whether this is done in sensor space, or in anatomical space following a process of source reconstruction, the resulting topology is again broadly consistent with that of other anatomical and functional brain networks (Stam & van Straaten, 2012). Furthermore, twin studies have shown that the small-world features of EEG networks present in various frequency bands are highly heritable (Smit, Stam, Posthuma, Boomsma, & de Geus, 2008).

Paediatric studies of EEG networks, however, suggest a different developmental trajectory towards these mature small-world networks than that observed in fMRI. For example, a large EEG study of 227 children recorded twice at 5 and 7 years of age found a shift from a more random configuration towards increased salience of small-world features, including increased clustering in the alpha band and increased path lengths in all frequency bands (Boersma et al., 2011). Similarly, a large cross-sectional study covering the age range 5–71 years showed increased clustering and path lengths, yielding increases in small-world coefficient, peaking around age 18 (Smit et al., 2012). This is in contrast to fMRI-based studies which do not robustly show changes in global network metrics over development but which qualitatively suggest that networks develop a more spatially distributed and topological integrative organization throughout childhood and adolescence. It is unclear whether these discrepancies between modalities are due to methodological differences (the EEG studies above, for example, were based on networks with nodes only in sensor space) or whether they suggest that early EEG activity and fMRI signal reflect different mechanisms of functional connectivity and therefore functional network topology (Fransson et al., 2013; Stam & van Straaten, 2012). We note that one recent EEG study of typically developing children between 2 and 6 years of age found that mean node degree, mean clustering coefficient and maximum betweenness centrality all increased with age, while the path length decreased in alignment with the idea of increasing functional integration observed in other imaging modalities (Bathelt, O'Reilly, Clayden, Cross, & de Haan, 2013). The study used source reconstruction with age-matched templates to construct EEG networks for 68 cortical regions of interest and did not control for age-related increases in the overall number of connections. A more complete exploration of the effect of various methodological decisions will likely be crucial in the future for resolving discrepancies both within and between modalities.

### Microanatomical and functional networks

It is worth remembering that a typical MRI voxel (1 mm^3^) represents a volume of grey matter that contains 35–70 million neurons (Pakkenberg & Gundersen, 1997). It is thus an outstanding challenge for neuroscience to develop methods than can link the organization and development of macronetworks measured by neuroimaging to the development of microscopic networks that are closer to cellular resolution. It is still early days in this endeavour but there are at least two experimental paradigms that lend themselves to studies of micronetwork development. One can analyse the development of a nervous system in vivo for a relatively simple organism like *C. elegans*; or one can analyse the development of networks in neuronal cultures in vitro.

#### Functional network development in neuronal cultures

As described above, correlated oscillations can be used to estimate macroscale functional connectivity and networks based on fMRI or EEG data. Likewise, correlated oscillations or bursting activity recorded from multielectrode arrays (MEAs) can be used to estimate microscale functional connectivity and networks in cultures of neurons (Blankenship & Feller, 2010). Spontaneous correlation patterns in neuronal activity have been observed not only in vivo, but also in cortical slices and even dissociated cell cultures (Marom & Shahaf, 2002). Neurons in dissociated cultures have been shown to spontaneously form microscopic networks whose activity, pharmacological response and various other properties seem fairly consistent with the same properties measured in comparable in vivo systems (Downes et al., 2012). Consequently, dissociated neuronal cultures are increasingly used in investigations of network organization and development (Downes et al., 2012; O'Connor, Huber, & Svoboda, 2009; Scanziani & Häusser, 2009).

These and other results are obtained by growing the culture on a multielectrode array (MEA). Each electrode (N∼100) of the MEA records a local field potential (LFP) that is analogous to macroscopic EEG signals in that it is not directly recorded at its (neuronal) source and many neurons may contribute to the LFP at each electrode. Functional networks are therefore often constructed in sensor space, based on correlations between the activity at each electrode. The neuronal systems typically studied with MEAs are small enough that individual neurons can potentially be resolved using microscopy or spike sorting algorithms, and manipulated using pharmacology or optogenetics (Mancuso et al., 2011). On the other hand, these systems are large enough that collective properties of neurons such as bursts, avalanches, oscillations and correlated activity can be studied. MEAs therefore offer a unique opportunity to study how neuronal mechanisms affect microscopic functional networks.

**Figure 3:**
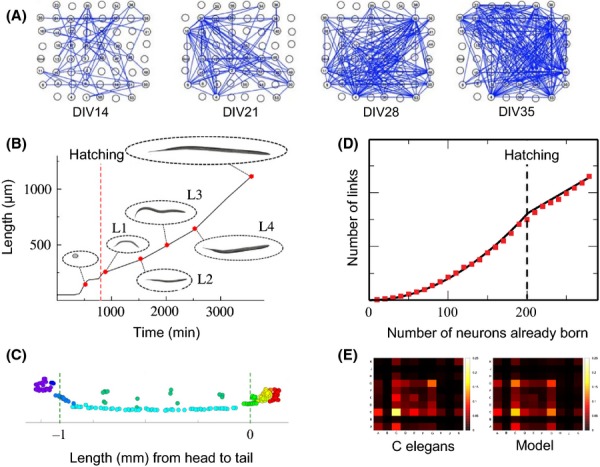
Microscopic network development. (A) The growth of a functional network in a neuronal culture at days in vitro (DIV) 14, 21, 28 and 35. The 8 by 8 grid corresponds to positions of the electrodes on the multielectrode array and edges are drawn between electrodes that have synchronized local field potentials (Downes et al., 2012). (B) Elongation schedule of the nematode *C. elegans* during development and (C) spatial distribution of neurons in the adult. These data were used to define the correct distance penalty to use when modelling the growth of its nervous system as an economical competition between minimizing connection distance (wiring cost) and maximizing the formation of high-degree hubs (Nicosia et al., 2013). This developmental model of a trade-off between biological cost and topological value was then able to capture several features of the system such as (D) the transition in growth rate observed at the time of hatching, and (E) the density of connections within and between anatomical groups of neurons – or ganglia – in the adult worm

Multielectrode array-based microfunctional networks have been shown to display similar topological characteristics to the macro-fMRI/MEG/EEG networks, including a parsimonious tendency for correlations to be short-range and clustered, as well as the existence of some long-range correlations ensuring the networks’ integrative capacity. MEA recordings from cultures of dissociated neocortical neurons have been used to study changes in the neuronal dynamics at different stages of development in the mouse (Egorov & Draguhn, 2013; Kamioka, Maeda, Jimbo, Robinson, & Kawana, 1996; Muramoto, Ichikawa, Kawahara, Kobayashi, & Kuroda, 1993). It is only recently, however, that graph theoretical analyses have been used explicitly to map the development of MEA-based functional networks (Chiappalone, Bove, Vato, Tedesco, & Martinoia, 2006; Downes et al., 2012). Downes et al. (2012) compared network properties in neuronal cultures at different stages of development during the first 5 weeks in vitro (see Figure3A). Starting at around 14 DIV (days in vitro), these networks spontaneously transitioned from an approximately random topology to an increasingly small-world organization, which stabilized at around 28 DIV. This process was accompanied by an increase in the number of hub nodes and a reduction in burst propagation time, consistent with the idea that small-worldness and the emergence of fat-tailed degree distributions are important for globally synchronized dynamics (Downes et al., 2012).

#### Growth of the *C. elegans* connectome

While MEAs provide an exciting model system for neuronal scale functional networks, the reconstruction of anatomical networks at the neuronal level has been a serious technical challenge. Serial reconstruction of electron microscopy sections can in principle be used, but is extremely laborious. In future, new optical microscopy techniques, such as CLARITY (Chung et al., 2013), may make it much simpler to accurately reconstruct anatomical connections between large numbers of neurons in vivo. However, to date, the nematode worm *C. elegans* is the only organism whose nervous system has been completely mapped at the cellular scale (White, Southgate, Thomson, & Brenner, 1986). Importantly, laser ablation studies of individual neurons have allowed biologists to assign functional roles to most neurons in the animal, making this system a valuable test-bed for network theoretic predictions about the functional role or importance of specific nodes or network features (Towlson et al., 2013).

The *C. elegans* connectome is also valuable in evaluating various hypotheses about the selection pressures that may drive network formation and growth. It is well recognized, as per Cajal's conservation principle, that many aspects of the *C. elegans* connectome are organized so as to approximately minimize the cost of connections between individual neurons (Chen et al., 2006; Cherniak, 1994; Cherniak, Mokhtarzada, Rodriguez-Esteban, & Changizi, 2004; Chklovskii, Schikorski, & Stevens, 2002). Subsequently, Kaiser et al. found that connections cost is not in fact strictly minimized and that it is possible to rewire the network, in silico, to obtain a somewhat reduced overall connection cost. They noted however that this reduction in wiring cost goes hand in hand with increased topological path length, making the network less efficient for information propagation (Kaiser & Hilgetag, 2006). It has also been shown that behaviourally important features of the network, such as a rich club of highly interconnected hubs (command interneurons that control coordinated movement of the animal), will incur more-than-minimal wiring cost (Towlson et al., 2013). Recent work has further explored this concept of an economical trade-off between biological cost and topological value as a generative principle of nervous system development.

Using data on the birth times (in minutes after fertilization) of each neuron, and the change in length of the organism as it grows from an egg through several larval stages to its adult form, Nicosia, Vértes, Schafer, Latora, and Bullmore (2013) tested several generative models of nervous system formation. The model that was found to most accurately reproduce several aspects of the system's growth and its adult configuration was an economical model that combined a form of preferential attachment term with a distance penalty (see Figure3B–E). Preferential attachment is a simple network growth rule, previously used to account for the formation of the world-wide web and other networks (Barabási & Albert, 1999), that stipulates that a new node making connections to the rest of the network will preferentially tend to form connections with hub nodes. The trade-off between this tendency for neuronal hubs to become progressively more central over the course of development, and the conservative or parsimonious drive to minimize wiring cost of a synaptic connection between neurons, provided a good generative model of several observed features of the development and adult organization of the *C. elegans* nervous system (Nicosia et al., 2013).

### Modelling normative brain network development

The computational strategies used to probe the organization and development of the *C. elegans* connectome are also increasingly being extended to advance our understanding of human brain development. One broad theme is the exploration of the costs and benefits of various topological network properties observed in the human connectome. Recent studies have, for example, uncovered the evolutionary advantages of modular organization in the face of variable environments (Kashtan & Alon, 2005), the propensity of small-world properties to support complex (Sporns, Tononi, & Edelman, 2000) and critical dynamics (Rubinov, Sporns, Thivierge, & Breakspear, 2011), and the role of hubs in the network's robustness to random attacks (Honey & Sporns, 2008; Kaiser, Robert, Andras, & Young, 2007).

**Figure 4:**
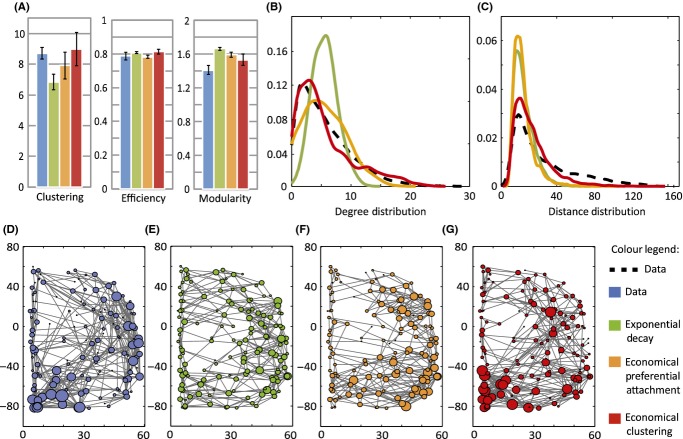
Comparison of networks simulated by three generative models with brain functional networks derived from experimental fMRI data on a group of 20 healthy volunteers (blue). The economical clustering model (red) yields significantly more realistic networks by all of the following measures: (A) Normalized clustering coefficient, global efficiency and modularity of brain functional networks. Degree (B) and distance (C) distributions. These are shown in solid coloured and dashed lines for the models and data respectively. (D–G) Schematic representation of the right hemisphere of the fMRI brain network for one participant (blue) and of a representative network generated by a single instantiation of each model. The size of each node represents the degree of the corresponding brain region

A second theme of interest has been the development of generative models to investigate which simple constraints (such as the need to minimize connection costs) are necessary and sufficient to drive the emergence of the observed brain network topology (Henderson & Robinson, 2011; Kaiser & Hilgetag, 2004a,b; Klimm, Bassett, Carlson, & Mucha, 2013; Vértes et al., 2012). Recently, we applied this generative modelling paradigm to human fMRI brain networks. In particular, we proposed an economical trade-off model in which the complex topology of brain networks emerges from two competing factors: (a) a parsimonious distance-penalty based on the cost of maintaining long-range connections, and (b) a bias, reminiscent of Hebbian learning rules, towards clustering or linking regions with a large amount of shared input (Vértes et al., 2012). Together, these two biologically plausible factors constrained the network to develop a range of topological properties characteristic of functional brain networks at both global and nodal levels of analysis (see Figure4). These results demonstrated how relatively simple generative models, representing biologically plausible processes, can provide a remarkably complete account of the complexity of human brain network organization.

Computational models can also be used in a variety of other ways to support mechanistic hypotheses for neurodevelopmental and neurodegenerative disorders. One recent example is the work by Zhou, Gennatas, Kramer, Miller, & Seeley (2012) showing that the pattern of atrophy for five different neurodegenerative diseases was consistent with spreading processes on the underlying brain functional networks. Importantly, modelling work also provides a much needed platform for testing the relationship between data observed at various scales and through various modalities. Neural mass models and more detailed spiking-network models involve simulating the activity of pools of neurons at each node of a large-scale anatomical network, with known anatomical connections providing coupling between the various local neuronal circuits. Such simulations have already been used to predict functional connectivity in the healthy adult human brain and are now being extended to a clinical setting (Cabral et al., 2013). Crucially, changes to cellular-level parameters or to the coupling strengths in this model will lead to quantifiable changes in functional connectivity which can be compared to the functional network phenotype of various disorders. In other words, such models offer opportunities to bridge the cellular and systems-level descriptions of brain disorders which are crucial to advancing their understanding and treatment.

## Neurodevelopmental disorders and brain networks

Many psychiatric and neurological disorders probably have their roots in abnormal brain development and have also been robustly associated with distributed grey and white matter deficits by case–control studies using whole-brain methods of mass univariate analysis like voxel-based morphometry (VBM). For many disorders, such as schizophrenia, ADHD and autism, imaging evidence of abnormal functional and anatomical connectivity has been added by multivariate or systems-level modelling of neuroimaging data. There is thus strong prior evidence that many brain disorders are both associated with abnormal brain networks and pathogenetically explicable in terms of abnormal brain development. We therefore expect to find evidence of abnormal brain network development in the emergence of neuropsychiatric disorders and their subsequent clinical progression or natural history. However, almost all graph theoretical studies of network organization in clinical disorders to date have used a case–control design, comparing a group of (often adult) patients to a comparison group of healthy volunteers. This design, although relatively simple to execute, does not directly provide developmental insights to the same extent as longitudinal designs comprising more than one measurement per participant. It is beyond the scope of this article to provide comprehensive reviews of all the relevant case–control studies in all disorders [see for example (Fornito & Bullmore, 2014) for recent more detailed reviews]. Here, we will focus on a few conceptual generalizations, drawing mainly from prior work on connectomics in schizophrenia.

First, it is notable that the ‘classical’ global topological metrics, such as characteristic path length or global efficiency, clustering and small-worldness, have been most widely applied in clinical studies to date; but they seem unlikely to be the most sensitive measures of diagnostic status or symptom severity. In some studies, including work on comatose patients scanned following massive acute brain injury, global topology of fMRI networks was remarkably well conserved despite the obvious clinical abnormality of the patients (Achard et al., 2012). In schizophrenia, on the other hand, it has been consistently reported that resting state fMRI networks have reduced clustering, conserved or slightly increased efficiency, and therefore reduced small-worldness. This pattern of results is compatible with the idea that network configuration in schizophrenia is shifted in the direction of greater randomness. However, similar evidence for subtle randomization of global network topology has been reported for many other disorders and indeed proposed as a hallmark of brain disorders in general (Stam & van Straaten, 2012).

In seeking to identify more sensitive and specific network markers of brain disorders, it may be useful to focus more on nodal properties, such as degree or hubness, and related aspects of the network community structure. High-degree nodes or hubs have been implicated in case–control studies of many brain disorders, including Alzheimer's disease and other neurodegenerative disorders (Buckner, Sepulcre, Talukdar, & Krienen, 2009; Seeley et al., 2009). Indeed, it was shown by meta-analysis of case–control VBM studies representing primary data on more than 20,000 patients with one of 26 clinical brain disorders, that grey matter ‘lesions’ measured by VBM were significantly more likely to occur in brain regions that were high-degree hubs in the normal structural or functional connectome (Crossley et al., 2014). Hubs are theoretically of special importance for understanding brain disorders for two, convergent reasons. First, the central topology and greater biological cost of hubs may make them more vulnerable to diverse disease processes. For example, the higher glucose metabolic rate of high-degree brain regions may make them disproportionately exposed to the pathogenic effects of hypoxia, ischaemia or oxidative stress. Second, the topologically valuable role of hubs in mediating high global efficiency of brain networks, which is likely to be crucial for integrated processing and ‘higher order’ cognitive functions, suggests that a lesion to a hub is more likely to generate neuropsychiatric symptoms of disordered mental function.

However, to develop a comprehensive network model of a developmental disorder will entail understanding how the disorganization evident in adult networks could have emerged from abnormal developmental processes over several years prior to clinical diagnosis. There are limited examples of how the developmental dimension can be incorporated in network studies of schizophrenia. The economical trade-off models introduced earlier to simulate normal fMRI network statistics can also be used to simulate the abnormal functional network statistics of people with schizophrenia. In this way, it was shown that accurate simulation of network organization in schizophrenia required a rebalancing of the trade-off between topological clustering and physical distance, so that the probability of a connection between regions was somewhat less penalized by the distance between them in schizophrenia (Vértes et al., 2012). This modelling result is consistent with some prior data showing that fMRI and structural covariance networks in schizophrenia have proportionally more long-distance connections than normal (Alexander-Bloch et al., 2012; Bassett et al., 2008).

**Figure 5:**
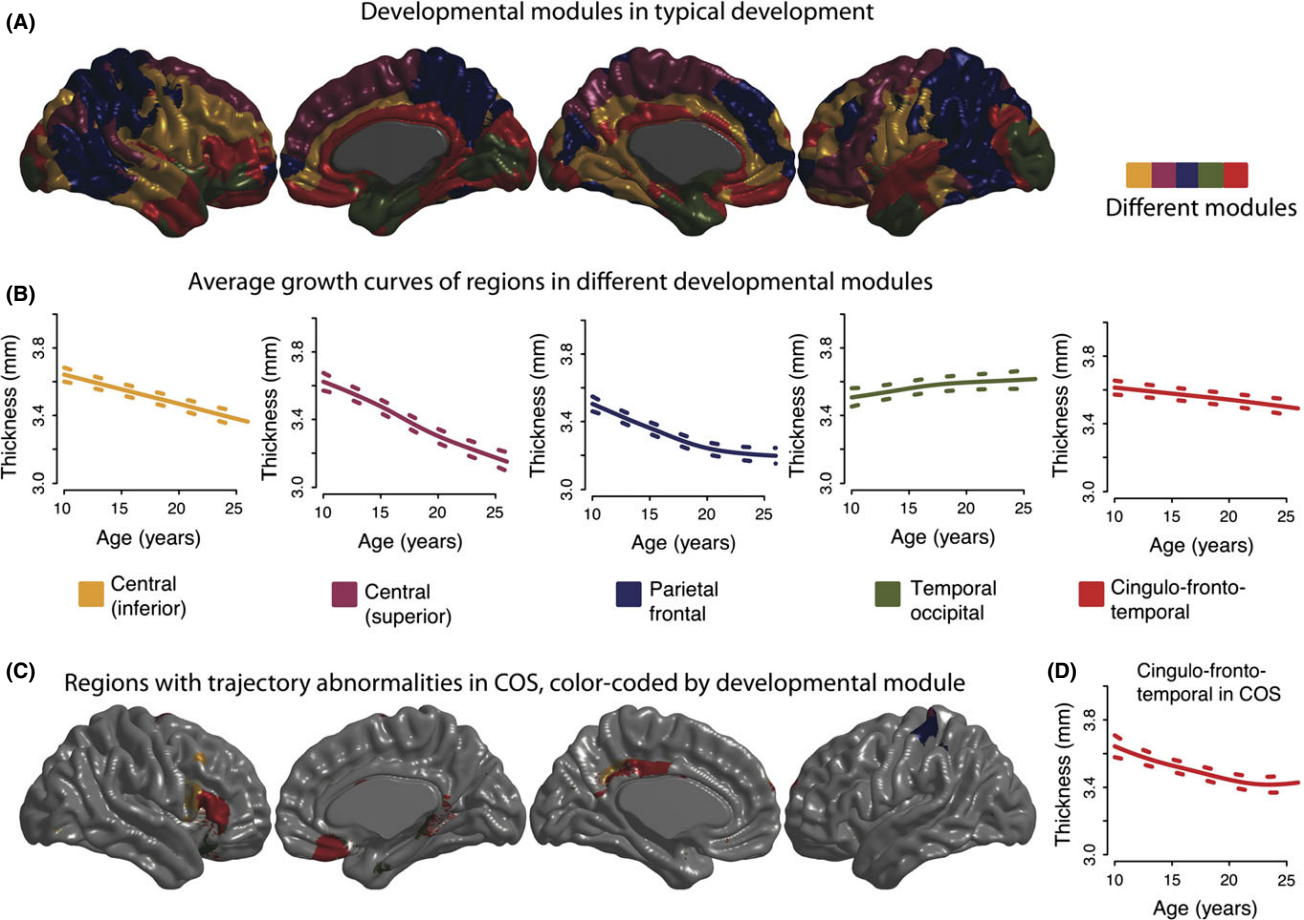
Developmental modules based on regional similarities in maturational trajectories, reproduced from Alexander-Bloch et al., (2014). In this study of typical development and childhood-onset schizophrenia (COS), 525 high-resolution magnetic resonance imaging scans were acquired on 208 subjects, 102 with COS. For each scan, thickness was estimated at 80,000 cortical vertices and penalized splines were used to estimate maturational trajectories (thickness as a function of age). (A–B) Using only healthy subjects, developmental modules were derived by clustering vertices with similarly shaped maturational trajectories. (C) Using both healthy subjects and subjects with COS, schizophrenia-related alterations in cortical maturation were tested for all cortical vertices. Finally, it was determined whether schizophrenia-related alterations in maturational trajectories were influenced by the organization of normative developmental modules

Although such results of generative modelling may suggest possible mechanisms of abnormal network growth, they do not detract from the need for more longitudinal studies. For example, Alexander-Bloch et al. (2014) used a large MRI data set on patients with childhood-onset schizophrenia (COS) to show that patients had local abnormalities of cortical thinning during adolescence that were concentrated in a single module of the normal maturational network (introduced above). Specifically, regions showing accelerated rates of cortical thinning in COS were located in a fronto-cingulo-temporal module of normally synchronized cortical maturation (see Figure5). This result provides proof of concept that the pattern of cortical abnormalities evident in patients with an established diagnosis may be constrained by the large-scale network organization of normal brain development in adolescence.

We note that in addition to these and other graph theoretical studies of schizophrenia there is a smaller but growing literature on topological analysis of structural and functional networks in other neurodevelopmental disorders. In autism, for example, widespread disruption of connectivity has been observed in both structural (Aoki, Abe, Nippashi, & Yamasue, 2013; Dziobek, Bahnemann, Convit, & Heekeren, 2010; McAlonan et al., 2005; Zhou, Yu, & Duong, 2014) and functional (Anagnostou & Taylor, 2011; Peters et al., 2013) brain networks. These abnormalities were found to predominantly affect long-distance connections (Aoki et al., 2013) and brain regions related to social cognition and other functions which are abnormal in autism (Dziobek et al., 2010; McAlonan et al., 2005). Similarly, convergent evidence from multiple modalities points to disrupted anatomical and functional connectivity, including higher clustering and lower global efficiency, of brain networks in ADHD (Cao et al., 2014; Konrad & Eickhoff, 2010). These abnormalities were found to be correlated with behavioural symptoms such as impulsivity and hyperactivity (Cao et al., 2014). Importantly, there have also been some studies seeking to compare connectomes between different developmental disorders (Di Martino et al., 2013) which will be a significant line of enquiry in future as we seek to define more clearly the diagnostic specificity of network markers.

## Future issues and conclusions

There are a number of important issues arising from the current state of the field of growth connectomics. The small sample sizes and methodological limitations of individual studies highlight the need to take an integrated view of developmental data across various neuroimaging modalities and analysis methods (Hagmann et al., 2012). In doing so, however, it is important to keep in mind the complex relationships between the information captured by these various modalities. For example, it is clear from both experimental and computational work that structural networks set strong constraints on the functional interactions that can occur in the network (Honey et al., 2009). However, as the pattern of large-scale anatomical connectivity is thought to change little beyond the age of 2 (Low & Cheng, 2006; Luo & O'Leary, 2005), the widespread changes in functional connectivity evident in later childhood and adolescence are likely caused by other, smaller scale developmental processes. Intriguingly, a recent study combining fMRI and diffusion imaging techniques has shown a clear increase in structure–function correlation with age (*R* = .74, *p* < .005) between 2 and 18 years (Hagmann et al., 2010). This is consistent with a growing number of other reports showing that structure–function relationships strengthen with development (Gordon et al., 2011; Supekar et al., 2010; Uddin et al., 2011).

Where results across modalities are found to be discrepant, it is difficult to know whether this reflects additional biological information (as the various modalities are in fact measuring different things) or whether it is indicative of some limitations and artefacts of imaging or analysis (for a recent review of outstanding technical issues see Hagmann et al., 2012). To more firmly establish the trajectories of brain network development, future studies could usefully include increased sample sizes and more longitudinal designs with two or more repeated measurements on each participant; multiple MRI modalities used to measure whole-brain structure and function in the same participants; and more refined measurement of cognitive scores whose age-related change can be correlated with the observed processes of brain network reorganization. In adults, both anatomical and functional network properties have already been linked to behavioural measures such as IQ or task performance (van den Heuvel et al., 2009; Seeley et al., 2007), and extending such results to a developmental setting will be central to our understanding of development.

Another question of clinical relevance is the mapping of normal gender differences in trajectories of brain network development. Virtually, all neuropsychatric disorders have different prevalence and symptomatology between males and females (Giedd & Rapoport, 2010) and morphological metrics such as grey matter volume (Lenroot et al., 2007) or cortical thickness (Sowell et al., 2007) have been found to show robust differences in developmental trajectory. Some studies have reported gender differences in graph theoretical measures of brain connectivity (Gong et al., 2009; Gong, He, & Evans, 2011; Yan et al., 2011; Dennis et al., 2013). While these generally point to higher connectivity and shorter path lengths in females, not all reports have been consistent (Dennis et al., 2013) and better powered developmental studies should shed light on this interesting area.

Taken together, various modalities of neuroimaging have revealed a broadly, but not entirely, convergent picture of human brain network development. Overall, networks seem to be topologically complex at birth, but their organization gradually evolves over childhood and adolescence from a local architecture dominated by sensory and sensorimotor areas to a more diffuse topology, facilitating higher level integrative functions. At the microscopic level, these network changes are thought to be underpinned by two key processes. First, synaptic pruning which is invisible to diffusion imaging but which potentially contributes to local decreases in functional connectivity and to the decreases in grey matter volume observed with structural MRI. Second, progressive myelination of long-range connections, which contributes to increased anatomical connectivity in DTI data as well as increased white matter volume in structural MRI, and the emergence of correlational structure over longer distances in functional MRI (Fornari, Knyazeva, Meuli, & Maeder, 2007; Gordon et al., 2011; Hagmann et al., 2012). More recently, several lines of evidence have converged to suggest that synaptic plasticity also plays a key role in defining functional connectivity (Vogel et al., 2010). For example, modest amount of training in adults was seen to increase functional connectivity between regions (Lewis, Baldassarre, Committeri, Romani, & Corbetta, 2009; Stevens, Buckner, & Schacter, 2010; Tambini, Ketz, & Davachi, 2010). However, there are obvious limitations to the directness and specificity with which cellular mechanisms of network development can be explored in humans. Thus, another important future trend is likely to be the greater use of animal MRI to map large-scale network development in other species, and to link these processes more directly to underlying maturational processes at a cellular level.

We conclude that growth connectomics has made a promising start in describing the developmental formation of complex brain networks in humans, and the ways in which brain networks can be abnormally in patients with neurodevelopmental disorders. There is still much to do in formulating a more coherent and comprehensive picture of large-scale brain network development and how the connectomic phenotype is linked to brain regional abnormalities, to cognitive function and to cellular mechanisms of development. Encouragingly, the general applicability of graph theoretical methods to diverse neuroscience data sets opens up the opportunity to explore network development in computational, cellular and animal models and ultimately, perhaps, to discover general principles of normal and abnormal network development at macro- and microscales.

**Key points**

- Due to the general applicability of graph theoretical methods to diverse neuroscience datasets, growth connectomics has made a promising start in describing the developmental formation of complex brain networks in humans.
- In this review, we integrate results from all macroscopic neuroimaging modalities, include insights gained from microscopic neuronal networks, and emphasize the potential driving forces shaping brain networks into their observed organization.
- We also briefly review the clinical relevance of network metrics as potential diagnostic markers and some recent efforts in computational modelling of brain networks in both health and disease.
- Overall, the body of work reviewed reveals the broadly, but not entirely, convergent picture of brain network development from a local architecture dominated by sensory areas to a more diffuse topology, facilitating higher level integrative functions.
- Future studies could usefully include increased sample sizes and more longitudinal designs with two or more repeated measurements on each participant; multiple MRI modalities used in the same participants; and more refined measurement of the age-related changes in cognitive scores.
